# Functional Dissection of the Multi-Domain Di-Heme Cytochrome *c*_550_ from *Thermus thermophilus*

**DOI:** 10.1371/journal.pone.0055129

**Published:** 2013-01-31

**Authors:** Sylvain Robin, Marzia Arese, Elena Forte, Paolo Sarti, Olga Kolaj-Robin, Alessandro Giuffrè, Tewfik Soulimane

**Affiliations:** 1 Chemical and Environmental Science Department, Materials and Surface Science Institute, University of Limerick, Limerick, Ireland; 2 Department of Biochemical Sciences and Istituto Pasteur – Fondazione Cenci Bolognetti, Sapienza University of Rome, Rome, Italy; 3 Consiglio Nazionale delle Ricerche Istituto di Biologia e Patologia Molecolari, Rome, Italy; Instituto de Tecnologia Quimica e Biologica, Portugal

## Abstract

In bacteria, oxidation of sulfite to sulfate, the most common strategy for sulfite detoxification, is mainly accomplished by the molybdenum-containing sulfite:acceptor oxidoreductases (SORs). Bacterial SORs are very diverse proteins; they can exist as monomers or homodimers of their core subunit, as well as heterodimers with an additional cytochrome *c* subunit. We have previously described the homodimeric SOR from *Thermus thermophilus HB8* (SOR_TTHB8_), identified its physiological electron acceptor, cytochrome *c*_550_, and demonstrated the key role of the latter in coupling sulfite oxidation to aerobic respiration. Herein, the role of this di-heme cytochrome *c* was further investigated. The cytochrome was shown to be composed of two conformationally independent domains, each containing one heme moiety. Each domain was separately cloned, expressed in *E. coli* and purified to homogeneity. Stopped-flow experiments showed that: i) the N-terminal domain is the only one accepting electrons from SOR_TTHB8_; ii) the N- and C-terminal domains are in rapid redox equilibrium and iii) both domains are able to transfer electrons further to cytochrome *c*_552_, the physiological substrate of the *ba*_3_ and *caa*_3_ terminal oxidases. These findings show that cytochrome *c*_550_ functions as a electron shuttle, without working as an electron wire with one heme acting as the electron entry and the other as the electron exit site. Although contribution of the cytochrome *c*_550_ C-terminal domain to *T. thermophilus* sulfur respiration seems to be dispensable, we suggest that di-heme composition of the cytochrome physiologically enables storage of the two electrons generated from sulfite oxidation, thereof ensuring efficient contribution of sulfite detoxification to the respiratory chain-mediated energy generation.

## Introduction

In addition to its natural occurrence in the environment, sulfite is an extremely important intermediary in sulfur metabolism, arising from a variety of reactions both in prokaryotes and eukaryotes [1]–[3]. The nucleophilicity and strong reducing capacity of sulfite account for its high toxicity. In the cell it can react with disulfide bonds causing protein inactivation and DNA damage. Although some microorganisms use sulfite as the sole electron/energy source [4], [5], accumulation of sulfite in the cell generally leads to massive damage, so that both prokaryotic and eukaryotic cells require efficient sulfite detoxification systems.

The most common strategy for sulfite detoxification in *Bacteria* and *Archaea* involves oxidation to sulfate, accomplished either directly or indirectly *via* the adenosine 5′-phosphosulfate reductase pathway [2], [6]. Molybdenum-containing sulfite:acceptor oxidoreductases (SORs) catalyze the direct oxidation of sulfite to sulfate. They have been identified in mammals [7], birds [8], plants [9] and prokaryotes [10]. Two types of SORs have been identified to date: i) the sulfite oxidases (EC 1.8.3.1) that are able to utilize O_2_ as a direct electron acceptor, but also ferricyanide and sometimes cytochrome *c*, and ii) the sulfite dehydrogenases (EC 1.8.2.1) unable to transfer electrons to O_2_. The first bacterial SOR was discovered almost half a century ago [11]. Although, since then, SORs have been shown to be widely distributed among bacteria, their exact physiological role is still elusive.

Compared to the vertebrate and plant enzymes, bacterial SORs are structurally much more diverse. The protein core consists of a molybdenum binding site and a dimerization domain. The enzymes can exist as monomers or homodimers of the core structure, as well as heterodimers with an additional cytochrome *c* subunit [10], [12]–[16]. The SORs containing both the molybdenum cofactor and the additional cytochrome *c* subunit have been classified as Group 1 SORs, while members of the Group 2 contain only the molybdenum cofactor and are called ‘atypical’ SORs [17]. The name ‘atypical’ arises from the fact that most of these enzymes, if not all, cannot efficiently use horse heart cytochrome *c* as substrate and display higher activities when assayed with the artificial electron acceptor ferricyanide. To date, Group 1 includes only the cytochrome *c-*containing SOR isolated from *Starkeya novella* [10]. The SOR from *Campylobacter jejuni*, though originally defined as a two-subunit protein acting similarly to the *S. novella* enzyme, likely belongs to Group 2 SORs, as the molybdenum- and the heme-containing subunits do not co-purify [18]. Similarly, all the other bacterial SORs so far described fall into Group 2 [12]–[16], thus calling for a revision of the term ‘atypical’.

With the exception of the enzyme from *Deinococcus radiodurans*, all characterized SORs were interestingly found to be encoded upstream their putative physiological electron acceptors (*c*-type cytochromes or other redox proteins [19]). Consistently, the genes coding for the *c*-type cytochromes identified as electron acceptors for SORs from *S. novella* [10], *C. jejuni* [18], *Sinorhizobium meliloti* [20] and *Thermus thermophilus* [16] were all found downstream the relative SOR-encoding gene. These *c*-type cytochromes are also very diverse. They differ in size and heme content and this is an additional feature contributing to the complexity of SORs.

Several attempts have been made to elucidate how SOR-mediated sulfite oxidation is integrated in cell metabolism. It is postulated that Group 1 SORs are directly linked to the respiratory chain *via* their natural electron acceptor cytochromes [20]. In *S. novella*, cytochrome *c*_550_ [10] was tentatively suggested to enable the association between sulfite oxidation and aerobic respiration, based on the notion that cytochromes *c* are natural substrates for cytochrome *c* oxidases [20]; the hypothesis however, remains to be tested as yet. Similarly, in *C. jejuni* electrons from sulfite oxidation were proposed to enter the respiratory chain downstream the *bc*_1_ complex *via* the natural substrate of *cb* oxidase [18]. Although such a scenario seems plausible, also in this case the exact electron transfer pathway and the redox proteins involved have not been identified. Only cell extracts, and not purified proteins, were used in these experiments and, indeed, one cannot exclude the involvement of additional, unidentified electron shuttles.

Recently, the connection to the respiratory chain was demonstrated for Group 2 SORs from *S. meliloti* [20] and *T. thermophilus* [16], whose natural electron acceptors (cytochrome *c* Smc04048 and cytochrome *c*_550_, respectively) have been identified. In the former study [20], however, experiments were performed using isolated cell membranes and the association of the electron acceptor of SorT, cytochrome *c* Smc04048, with cytochrome oxidases remains to be proven. On the contrary, in the latter study on *T. thermophilus* the complete electron transfer pathway linking sulfite oxidation to oxygen reduction was unveiled [16]. Accordingly, the electrons generated upon sulfite oxidation by SOR_TTHB8_ are transferred to the natural electron acceptor of the enzyme, cytochrome *c*_550_, and from here to cytochrome *c*_552_, the physiological electron donor of the two terminal cytochrome *c* oxidases, *ba*_3_ and *caa*_3_.

Here, the role of *T. thermophilus* cytochrome *c*_550_ in coupling sulfite oxidation to cell respiration has been further investigated.

## Materials and Methods

### Purification of Cytochrome c_552_ and the Cytochrome c Oxidases ba_3_ and caa_3_

Native *ba*_3_- and *caa*_3_-type cytochrome *c* oxidases were isolated from *T. thermophilus* HB8 cells according to previously published procedures [21], [22]. Native cytochrome *c*_552_ was purified according to Soulimane and co-authors [23]. Purified proteins were concentrated by ultrafiltration, fast frozen in liquid nitrogen and stored at −80°C.

### Expression and Purification of SOR_TTHB8_ and Cytochrome c_550_

Expression and purification of the proteins were conducted as described previously [16].

### Determination of Cytochromes and Cytochrome Oxidases Concentration

UV/vis absorption spectra were recorded with a Perkin Elmer Lambda 5 spectrophotometer. Concentration of the proteins was obtained from the dithionite reduced-minus oxidized spectra using the following extinction coefficients: ε = 18000 M^−1^ cm^−1^ (λ = 550 nm) for cytochrome *c*_550_; ε = 21000 M^−1^ cm^−1^ (λ = 552 nm) for cyt *c*_552_; ε = 6300 M^−1^ cm^−1^ (λ = 613 nm) for *ba*_3_ oxidase and ε = 24000 M^−1^ cm^−1^ (λ = 604 nm) for *caa*_3_ oxidase. The concentration of *SOR_TTHB8_* was determined using ε = 67350 M^−1^ cm^−1^ (λ = 280 nm).

### Limited Proteolysis

Cytochrome *c*_550_ was subjected to limited proteolysis by trypsin (Sigma-Aldrich) for 90 minutes at 37°C in 25 mM Tris-HCl pH 8.2, at a cytochrome:trypsin mass ratio of 100∶1. To separate the proteolytic products, the reaction mixture was diluted with water and loaded on a Fractogel® TMAE 650(S) column (Merck, Germany) equilibrated with 5 mM Tris-HCl pH 8.2. The flowthrough was collected, the column washed with the equilibration buffer and finally bound proteins were eluted with the same buffer containing 150 mM NaCl.

### Construction of the Cytochrome c_550_ Domains Expression Plasmids

The sequence encoding the mature N-terminal domain of cytochrome *c*_550_ (*c*_550_[N]) was amplified from *T. thermophilus* HB8 genomic DNA by PCR using the primers 5′–ATCTGACCATGGCTCAGACCACCCTCCCCGAG–3′, containing *Nco*I restriction site (underlined), and 5′–CAGTGACTCGAGTCAGGCAGGGGTCTCCTGGGCTG–3′, containing *Xho*I restriction site (underlined). Similarly, the sequence encoding the mature C-terminal domain of cytochrome *c*_550_ (*c*_550_[C]) was amplified using the primers 5′–ATCTGACCATGGCTCCCAAAACGGGAGCCCAGGTCTAC–3′, containing *Nco*I restriction site (underlined), and 5′–CAGTGACTCGAG TCATGGCAGTTTGAGGCCTTGGCGGAG–3′, containing *Xho*I restriction site (underlined). The products were *Nco*I and *Xho*I cloned into the expression vector pET22b+ (Invitrogen) to yield the pET22bC550N and pET22bC550C vectors. These constructs permit the expression of the recombinant domains in *E. coli*, fused to the *pelB* leader sequence for an optimal translocation to the periplasmic space.

### Expression and Purification of the Recombinant Cytochrome c_550_ N-terminal Domain (c_550_[N])

The BL21(DE3) *E. coli* strain was co-transformed with the pET22bC550N and pEC86 vectors [24], the latter containing the cytochrome maturation gene cluster necessary for the production of cytochrome *c* in *E. coli* under aerobic conditions [25]. The recombinant *c*_550_[N] was produced by growing the cells in LB medium containing ampicillin (100 µg/ml) and chloramphenicol (34 µg/ml) at 37°C for 24 h under shaking, and without protein expression inducers. Periplasmic proteins were prepared from fresh biomass. Cells were washed in PBS buffer (20 mM phosphate, 135 mM NaCl, 1 mM KCl, pH 7.4) and spun down at 8000×*g* for 20 min at 4°C. The pellet was resuspended in 100 mM Tris-HCl pH 8 buffer containing 0.75 M sucrose. Osmotic shock was induced by slowly adding 2 volumes of ice chilled 1 mM EDTA. Following 10 min incubation at room temperature, spheroplasts were prepared by incubation with 1 mg/ml lysozyme for 45 min at room temperature under gentle shaking. Following the addition of 25 mM MgCl_2_, and 50 µg/ml DNaseI to reduce the viscosity of the extract, intact spheroplasts were removed by centrifugation at 8000×*g* for 10 min at 4°C. The supernatant containing the recombinant domain was extensively dialyzed against 5 mM Tris-HCl pH 8.0 and then loaded on a Fractogel® TMAE 650(S) (Merck, Germany) column equilibrated at 4°C with the same buffer. The protein was eluted with a gradient of NaCl (0–250 mM) and the fractions containing the protein were pooled, concentrated and desalted using a PD10 column (GE Healthcare, Germany) equilibrated with 5 mM Tris-HCl pH 8.0. The eluate was then loaded on a CaptoQ XL anion exchange column (GE Healthcare, Germany) equilibrated with the same buffer and eluted with a gradient of NaCl (0–150 mM). Fractions containing the domain were pooled, concentrated and finally purified by gel filtration on a Superdex 75 column (GE Healthcare, Germany) at 4°C with 5 mM Tris-HCl pH 8.0 buffer containing 150 mM NaCl. The isolated protein was concentrated by ultrafiltration, fast frozen in liquid nitrogen and stored at −80°C.

### Expression and Purification of the Recombinant Cytochrome c_550_ C-terminal Domain (c_550_[C])

The C-terminal domain was expressed and initially extracted as described above for the N-terminal domain. The supernatant containing the *c*_550_[C] domain was extensively dialyzed against 5 mM Tris-acetate buffer pH 6.0 and then loaded on a CM Sepharose® (Merck, Germany) column equilibrated at 4°C with the same buffer. The protein was eluted with a gradient of NaCl (0–250 mM) and the fractions containing the protein were pooled, concentrated and finally purified by gel filtration on a Superdex 75 column (GE Healthcare, Germany) at 4°C with 5 mM Tris-HCl pH 8.0 buffer containing 150 mM NaCl. The last step was repeated twice. The isolated protein was concentrated by ultrafiltration, fast frozen in liquid nitrogen and stored at −80°C.

### Analytical Size Exclusion Chromatography

Analysis of the association of cytochrome c*_550_* domains was carried out by analytical size exclusion chromatography (SEC). 1.3×10^−8^ moles of each domain were incubated for one hour at room temperature in 10 mM Tris-HCl, 50 mM NaCl, pH 8.0 before injection on a Superdex S75 10/30 column at a flow-rate of 0.5 ml/min. Elution profiles were recorded at 280 nm. The cytochrome full-length was analysed in a similar way.

### Electron Transfer activity

Stopped-flow experiments were carried out with a thermostated instrument (DX.17 MV, Applied Photophysics, Leatherhead, UK), equipped with a 1-cm pathlength observation chamber. Reactions were investigated by monitoring the absorption changes at selected wavelengths. When necessary, ionic strength was adjusted by addition of KCl and the buffer was degassed with vacuum/N_2_ cycles. Data were analyzed using the software MATLAB (The Mathworks, South Natick, MA).

Reduction by SOR_TTHB8_ of the cytochrome *c*_550_ and its domains was investigated anaerobically at 45°C in 100 mM Tris-HCl pH 8.0 buffer containing 0.1 mM EDTA. To prevent inhibition of SOR_TTHB8_ resulting from prolonged incubation of the enzyme with a large excess of sulfite, in these experiments the stopped-flow instrument was used in the sequential mixing mode. Typically, 2 µM SOR_TTHB8_ was pre-mixed with 4 mM sulfite and after 500 ms further mixed with increasing amounts of oxidized cytochrome *c*_550_, *c*_550_[N], *c*_550_[C] or a 1∶1 mixture of the two domains. The reduction of the cytochrome or its domains was monitored at 418 nm or, in the case of a too high signal in the Soret region, at 555 nm. The turnover rates (TN) of reduction of cytochrome *c*_550_ and its domains were calculated by dividing the concentration (expressed in µM) of the *c*_550_ sample reduced at t = t_½_ by the half time of the reaction and the concentration of SOR_TTHB8_ in the experiment (typically 0.5 µM after mixing).

The kinetics of electron transfer between ascorbate-reduced cyt *c*_550_ (or its domains) and cyt *c*_552_ was assayed anaerobically at 4°C. This low temperature was chosen to slow-down and thus better resolve in time the reactions. Experiments were carried out in 5 mM Bis-Tris pH 7.0 buffer under non-pseudo-first order conditions, i.e., at comparable concentrations of the two proteins. Observed rate constants (*k*_obs_) were therefore obtained by fitting the experimental time courses to the equations described in [26] for the analysis of bimolecular reactions assayed under second-order conditions.

The oxidation of cytochrome *c*_550_ and its domains by *ba*_3_- or *caa*_3_-oxidase was assayed at 25°C in 5 mM Bis-Tris pH 7.0 buffer complemented with 0.1% *n*-Dodecyl-β-D-maltoside. Approximately 1.5 µM *c*_550_, *c*_550_[C], *c*_550_[N] or a 1∶1 mixture of the two domains was pre-reduced with 300 µM ascorbate and stopped-flow mixed with air-equilibrated buffer containing either *ba*_3_- (200 nM) or caa_3_-oxidase (200 nM). The reaction was followed at 418 nm.

## Results

### The Cytochrome *c*_550_ is Composed of Two Conformationally Independent Domains

The recently identified periplasmic di-heme cytochrome *c*_550_ [16] as a whole shows poor similarity to known proteins. However, when analyzed separately, its N-terminal domain sequence containing one heme binding site presents a high homology to the subunit B of SOR from *C. jejuni* [18], whereas the C-terminal domain, containing the other heme binding site, exhibits a high sequence identity with *c*_552_ from *T. thermophilus* HB8. This led to the hypothesis that cytochrome *c*_550_ is likely organized in two distinct domains, each one possibly presenting an independent fold and a heme cofactor, with distinct roles in mediating electron transfer between SOR_TTHB8_ and the respiratory chain, through cytochrome *c*_552_ [16].

**Figure 1:**
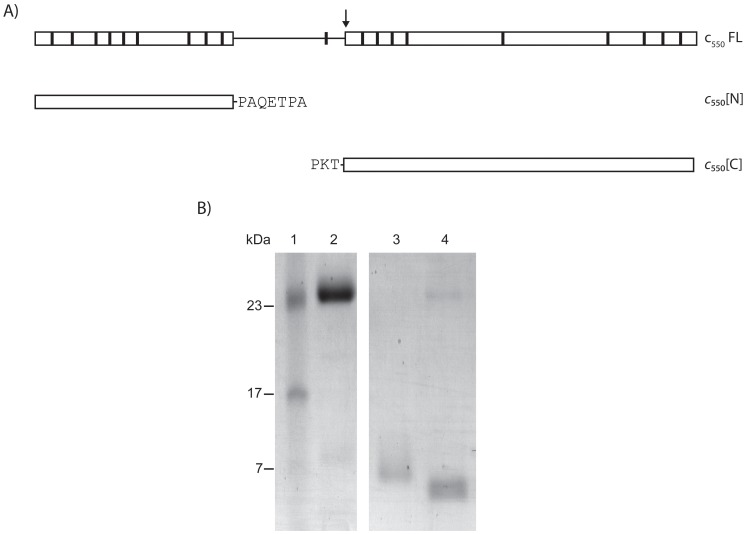
Domains of *T. thermophilus* cytochrome *c*_550_. The schematic representation of the full-length cytochrome *c*_550_ (FL) with predicted trypsin cleavage sites (vertical lines) and the cloned *c*_550_[N] and *c*_550_[C] domains is shown in panel A. The predicted boundary of the two domains is indicated by an arrow. Panel B shows the SDS-PAGE analysis of cytochrome *c*_550_ after limited proteolysis with trypsin and IEX chromatography (Fractogel® EMD TMAE(S)). Lanes: 1– molecular marker; 2– full-length cytochrome *c*_550_; 3– protein sample in the column flowthrough assigned to *c*_550_[C]; 4– protein sample eluting from the column at 150 mM NaCl assigned to *c*_550_[N].

Consistently, according to the DomPred server [27] cytochrome *c*_550_ consists of two domains, with a predicted boundary at residue 107 and a proline rich region (91–106 aa) likely representing a flexible inter-domain linker [28] (Figure 1A). While globular proteins, due to their native rigid structure, are typically resistant to proteolysis under physiological conditions, flexible inter-domain linkers can be substrates for proteases. This makes the limited proteolysis approach suitable to confirm the multi-domain organization of a protein [29]. Based on this notion, cytochrome *c*_550_ was subjected to limited proteolysis using trypsin. Overall, the cytochrome presents 19 putative cleavage sites for this protease (Figure 1A). Among these sites, the one in position K105 is of particular interest, as it is located within the predicted linker, being therefore potentially more accessible to the protease. On this basis, limited proteolysis of the recombinant cytochrome *c*_550_ is expected to yield the N- and C-terminal domains as the major cleavage products.

As *c*_550_[N] and *c*_550_[C] have significantly different calculated isoelectric points (5 and 7.97, respectively), the two domains are expected to have opposite net charges at pH 7, being easily separable by ion exchange (IEX) chromatography. The products of the limited proteolysis of cytochrome *c*_550_ were therefore directly subjected to analytical IEX chromatography using the basic anionic exchanger Fractogel® EMD TMAE (S) and subsequently analyzed by SDS-PAGE. As expected, two major products of limited proteolysis were obtained in addition to the band corresponding to non-digested cytochrome (Figure 1B). The bigger fragment in the flowthrough can be attributed to *c*_550_[C], while the shorter fragment eluting at 150 mM NaCl likely corresponds to *c*_550_[N]. This result confirms that the protein as a whole is strongly dipolar with a negatively charged *c*_550_[N] and a positively charged *c*_550_[C]. The apparent molecular weights of the proteolytic products (∼7 kDa and <7 kDa) are smaller than those predicted for *c*_550_[C] and *c*_550_[N] (14 kDa and 9 kDa, respectively). This is most likely due to the presence of multiple trypsin cleavage sites within the protein, especially those located at the termini of the domains, more easily accessed by the protease (Figure 1A). The limited proteolysis did not disturb the core of the domains or the heme binding, as the two generated fragments exhibited UV-Vis spectroscopic properties identical to those of the individually expressed domains (see below). This indicates that the two proteolytic fragments correspond to the two domains_,_ each of them being independently folded and associated with one heme cofactor.

**Figure 2:**
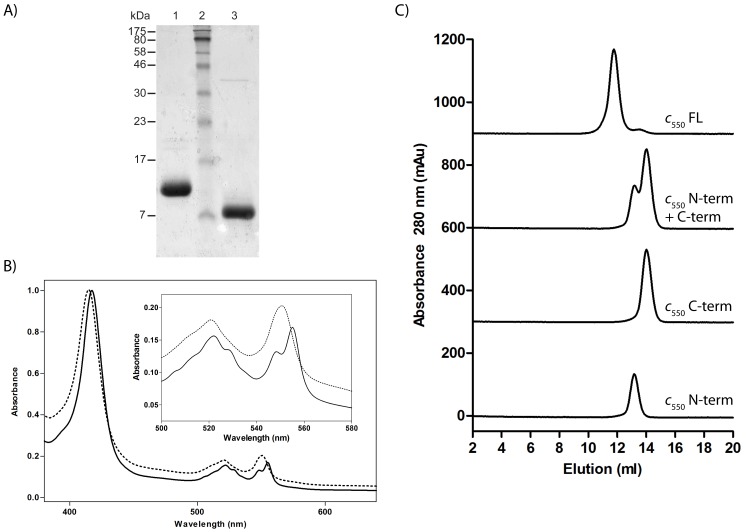
Characterization of the recombinant N- and C-terminal domains of cytochrome *c*_550_. The purified domains have been characterized by SDS-PAGE (A), absorption spectroscopy (B) and analytical SEC (C). A. Lanes: 1 and 3– purified *c*_550_[C] and *c*_550_[N], respectively; 2– molecular marker. B. Absorption spectrum of reduced *c*_550_[N] (–) and *c*_550_[C] (−). Inset: enlargement of the visible region. C. Analytical SEC chromatograms relative to (from top to bottom) the full-length cytochrome *c*_550_, a mixture of *c*_550_[C] and *c*_550_[N], and each of the two domains analyzed separately.

The use of recombinantly produced, isolated polypeptidic domains has proven to be a valuable approach to investigate intra- and intermolecular electron transfer between redox centers in multi-domain proteins with largely overlapping spectral properties [30], [31]. Therefore, to further confirm the existence of two independent domains in cytochrome *c*_550_ and to investigate their function, the N- and C-terminal parts of the protein were individually produced in *E. coli.* The recombinant domains contained the PelB signal sequence to promote their translocation to the periplasmic space and fragments of the flexible linker in order to enhance the stability of both domains (Figure 1A). After protein expression and isolation of the periplasmic fractions, the two domains were purified by IEX and size exclusion chromatography (SEC), as described in the Materials and Methods section. The procedure yielded ∼95% homogeneous fragments of approximately 9 kDa and 14 kDa corresponding to *c*_550_[N] and *c*_550_[C], respectively (Figure 2A). The UV-Vis spectra of the reduced *c*_550_[N] and *c*_550_[C] exhibited a Soret band centered at 415 nm and 417.5 nm, respectively, while *c*_550_[C] showed also a composite α band (Figure 2B). This shows that the splitting of the signal does not arise from the presence of two hemes. Instead, it is the result of the transition between the ground state and two or more excited states close in energy. Overall, the spectral properties confirmed that one heme cofactor was successfully incorporated in each domain during recombinant expression in *E. coli*.

Separation by IEX chromatography of the domains obtained by limited proteolysis of cytochrome *c*_550_ (Fig. 1B) argues against the formation of a stable complex between *c*_550_[N] and *c*_550_[C]. Consistently, when a 1∶1 mixture of the two domains was incubated at room temperature for one hour and assayed by analytical SEC, *c*_550_[N] and *c*_550_[C] eluted separately as individual proteins (Figure 2C). All together the results presented above indicate that cytochrome *c*_550_ folds into two independent domains with distinct properties, each carrying a single heme group. These features may have implications with regard to the electron transfer activity of the cytochrome.

### The N-terminal Domain of Cytochrome *c*_550_ Accepts the Electrons from SOR_TTHB8_

**Figure 3:**
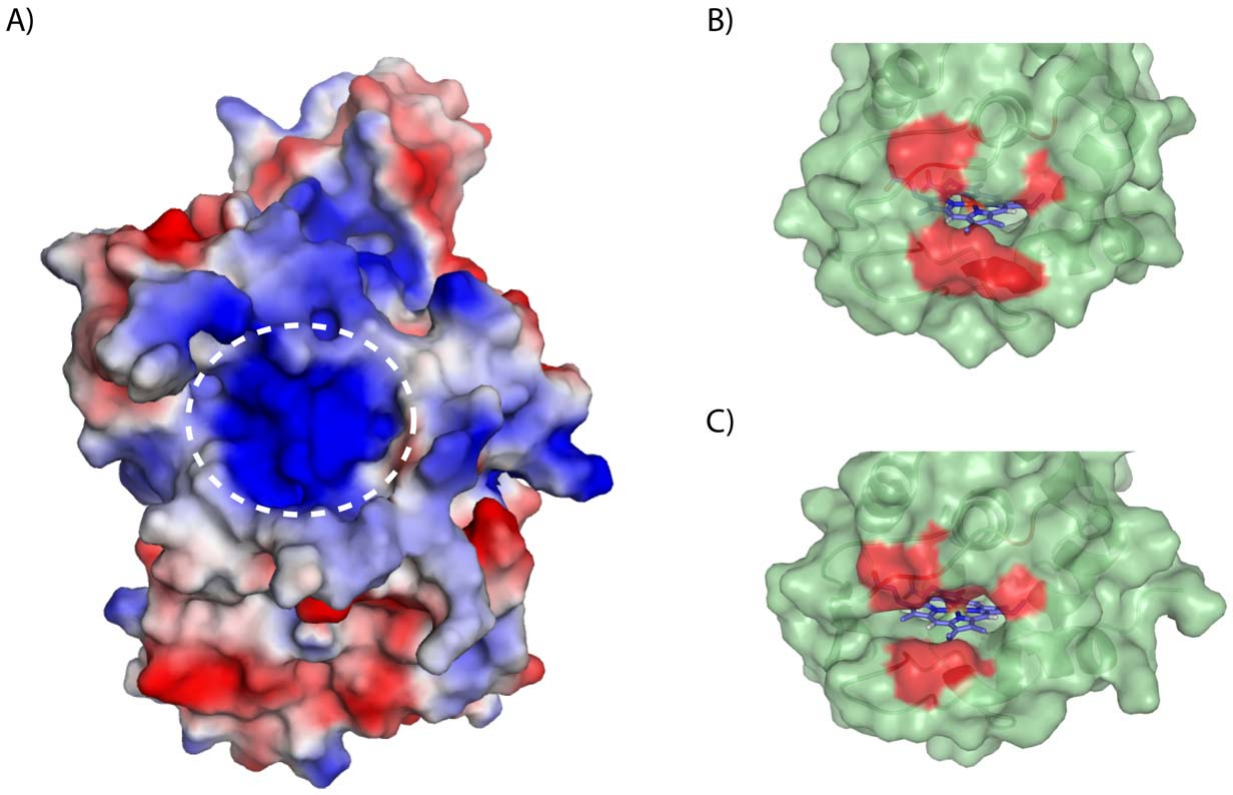
The 3D models of SOR_TTHB8_ (A) and cytochrome *c*_550_ (B), shown together with the structure of cytochrome *c*_552_ (PDB ID: 1c52) (C). Structure analysis of SOR_TTHB8_ revealed the positively charged surface area of the protein (A, encircled), believed to be important for interaction with cytochrome *c*_550_. The hydrophobic belt surrounding the heme cleft in cytochromes *c*_550_ (B) and *c*_552_ (C) is depicted in red.

We have previously shown that cytochrome *c*_550_ is the physiological electron acceptor of the sulfite:cytochrome *c* oxidoreductase encoded by the *ttha1326* gene in *T. thermophilus* HB8 [16]. Based on the high similarity between *c*_550_[N] and the SorB subunit of the sulfite:cytochrome *c* oxidoreductase from *C. jejuni*, it is very likely that this domain acts as an electron acceptor for SOR_TTHB8_. The 3D model of SOR_TTHB8_ was automatically built by means of the SWISS-MODEL server [32]–[34], using as a template the only available structure for a microbial SOR, i.e., the one of the *S. novella* enzyme (PDB ID: 2c9x, segment A; 33% identity to SOR_TTHB8_) (Figure 3A). In *S. novella* SOR, the formation of complementary electrostatic surfaces at the interface of the SorA and SorB subunit of sulfite:cytochrome *c* oxidoreductase has been observed [35]. Similarly, a positively charged surface area surrounding the pocket cradling the molybdopterin cofactor is present also in the SOR from *T. thermophilus* HB8 (Figure 3A), while the *c*_550_[N] presents an overall negative surface charge as shown by IEX chromatography. It is, therefore, reasonable to assume that *c*_550_[N] and SOR_TTHB8_ present analogies to typical SORs both in terms of function and type of interactions.

**Figure 4:**
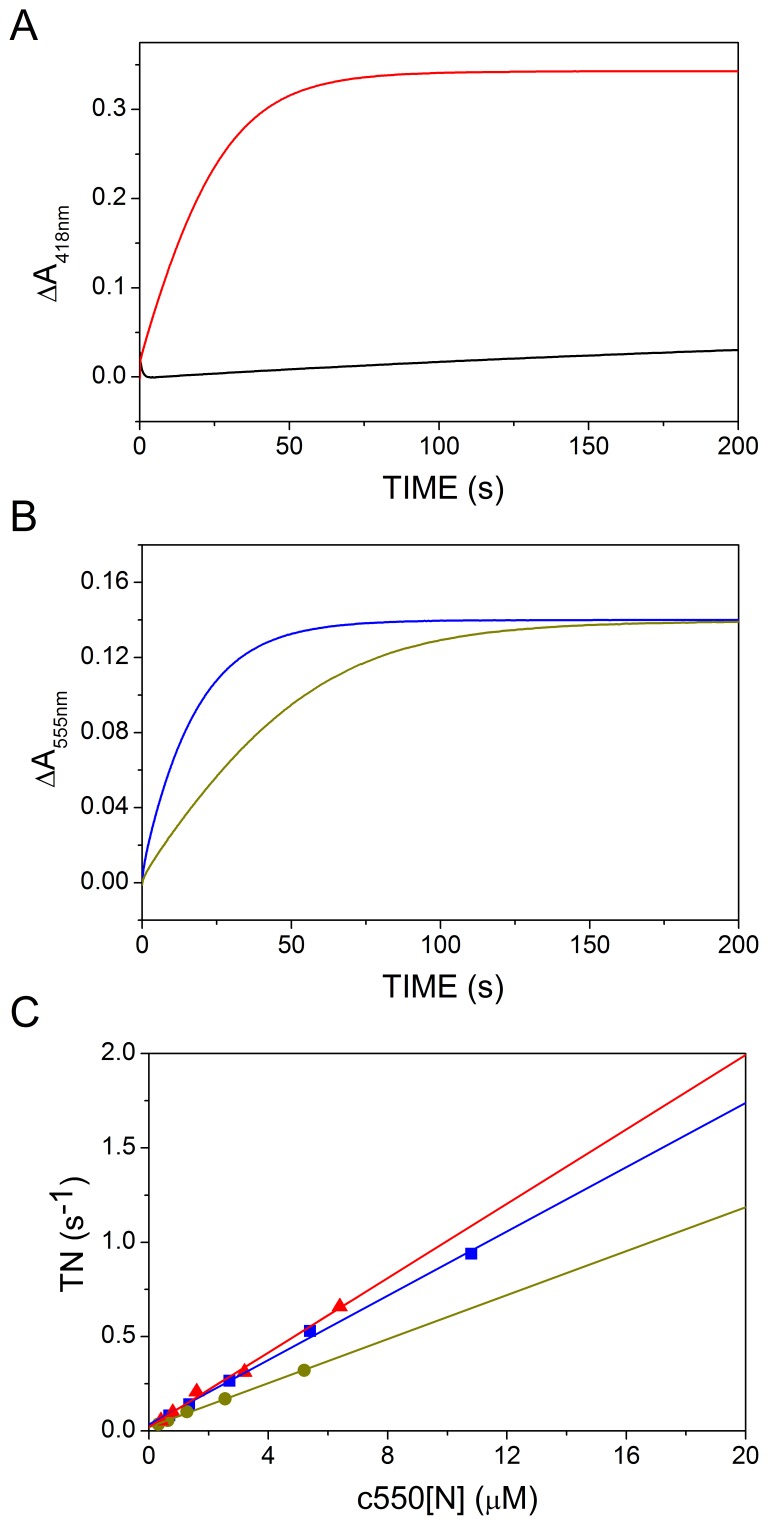
Reduction of cytochrome *c*_550_ and its domains by SOR_TTHB8_ in the presence of sulfite. In order to reveal the domain of *c*_550_ accepting electrons from SOR_TTHB8_, the reduction of full length *c*_550_ (3.2 µM, red), *c*_550_[C] (2.5 µM, black), *c*_550_[N] (10.3 µM, blue) or a 1∶1 mixture of *c*_550_[N] and *c*_550_[C] (5.15 µM each, green) by 0.5 µM SOR_TTHB8_ in the presence of 1 mM sulfite was performed (A, B). The experiments were carried out at T = 45°C. Panel C shows the turnover rates (TN) for *c*_550_ (red), *c*_550_[N] (blue) and a mixture of *c*_550_[N] and *c*_550_[C] (green), calculated as described in Materials and Methods.

In order to test which domain of cytochrome *c*_550_ preferably interacts with SOR_TTHB8_, the electron transfer between the latter enzyme and the recombinant *c*_550_[N] or *c*_550_[C] was kinetically investigated by stopped-flow spectroscopy. In these experiments, a solution of SOR in the presence of an excess of sulfite was anaerobically mixed at 45°C with increasing amounts of oxidized full-length cytochrome *c*_550_, *c*_550_[N], *c*_550_[C] or a 1∶1 mixture of the two domains. As shown in Figure 4A, compared to the full-length protein, *c*_550_[C] acts as a very poor electron acceptor for SOR_TTHB8_ even at a final concentration as high as 20 µM (not shown). In contrast, *c*_550_[N] is promptly reduced in this experimental set up (Figure 4B), which confirms that the N-terminal domain of cytochrome *c*_550_ is the electron acceptor for SOR_TTHB8_. Interestingly, a 1∶1 mixture of the two cytochrome domains is also completely reduced, although at a slightly lower apparent rate (Figure 4B). This implies that *c*_550_[N] and *c*_550_[C] are in rapid redox equilibrium and can exchange electrons once reduction of the *c*_550_[N] has occurred. Figure 4C reports the average turnover rate of the reaction calculated as detailed in the Materials and Methods and plotted as a function of the concentration of *c*_550_[N], either isolated or as part of the full-length protein or mixed in a 1∶1 ratio with *c*_550_[C]. Under all conditions a linear concentration dependence was observed. Interestingly, *c*_550_[N], either isolated or integrated in the full-length protein, is reduced at comparable rates, which further points to the N-terminal domain of cytochrome *c*_550_ as the electron acceptor for SOR_TTHB8_.

### Electron Transfer from Cytochrome *c*_550_ to Cytochrome *c*_552_

**Figure 5:**
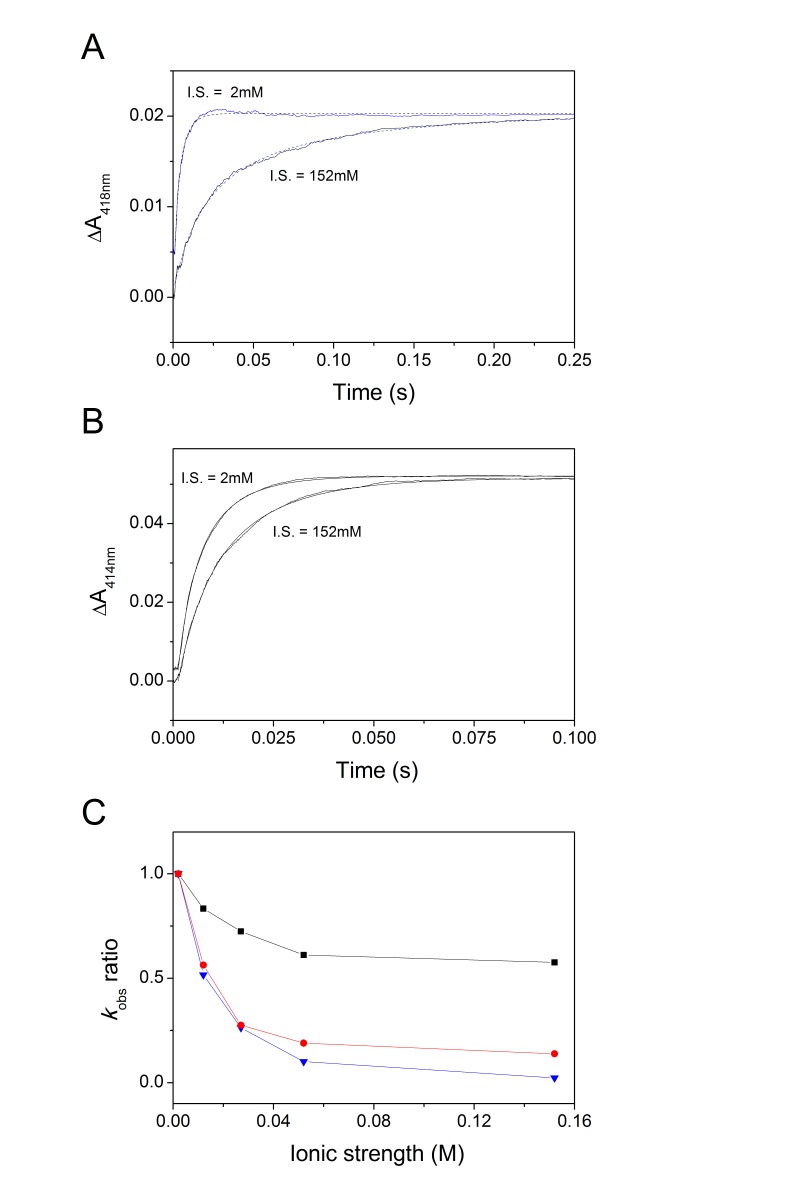
Electron transfer analysis between *c*_550_[N], *c*_550_[C] and *c*_552_. To investigate the electron transfer between *c*_550_[N], *c*_550_[C] and *c*_552,_ kinetic traces were collected after anaerobically mixing 3.2 µM ascorbate-reduced *c*_550_[N] with 3.2 µM oxidized *c*_552_ at λ = 418 nm and T = 4°C (A), or mixing 4.1 µM ascorbate-reduced *c*_550_[C] with 5.2 µM oxidized *c*_552_ at λ = 414 nm and T = 4°C (B). Panel C shows the ionic strength dependence of the reaction of *c*_550_ (red), *c*_550_[N] (blue) or *c*_550_[C] (black) with *c*_552_. Data were normalized for the rate constants measured at ionic strength = 2 mM.

We have previously shown that cytochrome *c*_550_ passes the electrons generated during sulfite oxidation to the terminal oxidases of the respiratory chain *via* cytochrome *c*_552_. To assess which domain of cytochrome *c*_550_ takes part in this electron transfer, the oxidation of *c*_550_[N] or *c*_550_[C] by cytochrome *c*_552_ was tested. The reduced *c*_550_[N] or *c*_550_[C] were mixed anaerobically with oxidized *c*_552_ and the reaction was followed at 4°C at ionic strengths ranging from 2 to 152 mM. The reaction was studied under non pseudo-first order conditions and, accordingly, the experimental traces were fitted following the analysis described elsewhere [26]. From the results presented in Figure 5AB it can be concluded that, despite the low temperature, both *c*_550_[N] and *c*_550_[C] are able to rapidly exchange electrons with cytochrome *c*_552_. Table 1 shows the estimated forward (k_F_) and reverse (k_R_) rate constants of the reaction measured at the same ionic strength (12 mM). The observed differences in k_F_ and k_R_ are not significant due to the rather high experimental error in those measurements, partly arising from the large optical overlap among the investigated proteins. Based on the results, we conclude that both *c*_550_[N] and *c*_550_[C] exchange electrons with cytochrome *c*_552_ at rates similar to those previously measured with the full-length cytochrome *c*_550_ [16]. Interestingly, the reaction between *c*_550_[N] and *c*_552_ displays a similar ionic strength dependence to the full-length protein, whereas the dependence is less pronounced in the case of *c*_550_[C] (Figure 5C). This suggests that electrostatic forces play an important role in the interaction between *c*_550_[N] and *c*_552_, while apolar interactions may be involved in molecular recognition between *c*_550_[C] and *c*_552_.

**Table 1:** Forward (k_F_) and reverse (k_R_) rate constants estimated at 4°C and at ionic strength = 12 mM for the reaction of cytochrome *c*_550_ and its domains with cytochrome *c*_552_.

	*k*_F_ (M^−1^ s^−1^)	*k*_R_ (M^−1^ s^−1^)
*c*_550_[N]	∼10×10^7^	8 ÷ 30×10^6^
*c*_550_[C]	∼7×10^7^	3 ÷ 7×10^6^
c_550_	∼5.5×10^7^	5 ÷ 9×10^6^

In order to test this possibility, the 3D model of *c*_550_[C] was automatically built by means of the SWISS-MODEL server [32]–[34], using the X-ray structure of cytochrome *c*_552_ from *T. thermophilus* HB8 (PDB ID: 1c52; ∼50% identity) as template (Figure 3BC). The heme cleft in the very well described cytochrome *c*_552_ is surrounded by a hydrophobic belt (Figure 3C) consisting of residues G13, C14, F26, V68, M69 and F72; this patch of residues likely participates in the interaction between the cytochrome and the terminal oxidase *ba*_3_ [36]. Interestingly, the model structure of *c*_550_[C] clearly shows that such hydrophobic residues (G11, C12, F24, V66, M67 and F70) are structurally conserved around the cleft (Figure 3B). It is therefore likely that molecular recognition between *c*_550_[C] and *c*_552_ is also mediated by this hydrophobic patch, in line with the modest ionic strength dependence reported in Figure 5C.

### Electron Transfer to the Terminal Oxidases *caa*_3_ and *ba*_3_

**Figure 6:**
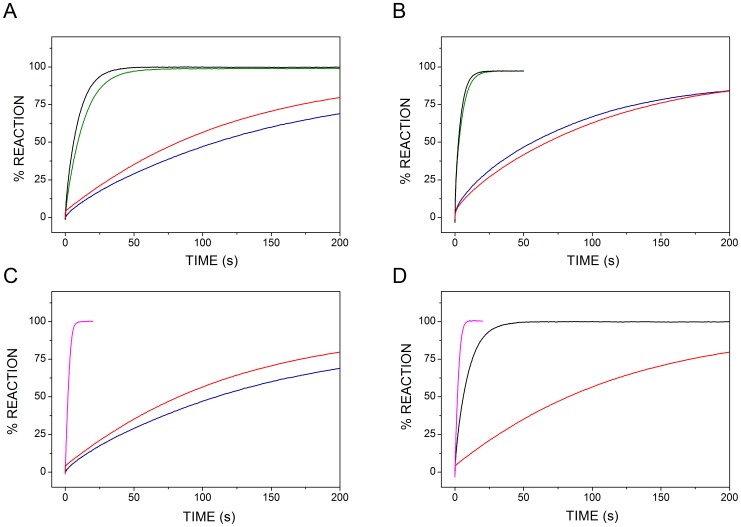
Electron transfer between the domains of *c*_550_ and terminal oxidases *ba*_3_ and *caa*_3_ in the absence (A,B) or in the presence (C, D) of cytochrome *c*_552_. Oxidation by *ba*_3_ (A) or *caa*_3_ (B) oxidase of ascorbate-reduced *c*_550_ (red), *c*_550_[C] (black), *c*_550_[N] (blue) or a 1∶1 mixture of the two domain (green). In C and D, the oxidation of *c*_550_[N] or *c*_550_[C] by *ba*_3_ oxidase was investigated in the presence of 10 nM *c*_552_ (magenta). T = 25°C.

When assayed separately, *c*_550_[N], similarly to full-length *c*_550_, is very slowly oxidized by either *ba*_3_ or *caa*_3_ oxidase, whereas *c*_550_[C] is quickly oxidized by either of the two oxidases (Figure 6A,B). Considering the high sequence similarity between *c*_550_[C] and *c*_552_ and the structurally conserved hydrophobic belt (Figure 3BC) likely participating in molecular recognition between *c*_552_ and *ba*_3_-oxidase, this finding is perhaps not unexpected. On the other hand, we have previously shown (and confirmed here) that the electron transfer between full-length *c*_550_ and *ba*_3_ or *caa*_3_ is not efficient, unless mediated by *c*_552_ acting as an electron shuttle [16]. Therefore, we suggest that the relatively fast direct electron transfer between *c*_550_[C] and terminal oxidases here documented is of no physiological value. If the conserved hydrophobic patch mentioned above is involved in mediating electron transfer from *c*_550_[C], in the full-length protein such a patch should be not accessible by large molecules, like *ba*_3_ or *caa*_3_ oxidase, though possibly still allowing interaction with much smaller molecules such as cytochrome *c*_552_.

Interestingly, a 1∶1 mixture of ascorbate-reduced *c*_550_[N] and *c*_550_[C] is also quickly and fully oxidized by *ba*_3_ or *caa*_3_ (Figure 6A,B), which further confirms that the two domains of *c*_550_ are in relatively fast redox equilibrium. Expectedly, in the presence of 10 nM oxidized *c*_552_, both *c*_550_[N] and *c*_550_[C] are promptly oxidized by *ba*_3_ (Figure 6C,D) or *caa*_3_ (data not shown), as previously observed with the full-length protein [16]. This further supports a fast electron transfer between either of the two domains of cytochrome *c*_550_ and cytochrome *c*_552_.

## Discussion

While sulfite oxidizing enzymes (SOEs) in vertebrates and humans have been studied for over 40 years, significant progress in studying bacterial SOEs has only been made in the last decade. Bacterial SOEs are extremely diverse in terms of structure and oxidizing proteins. Discovery of novel SOEs is being constantly reported, but in most cases the relative electron acceptor was not identified. Hence, usually the link to the respiratory chain is not shown [12]–[14], [18], [20]. The complexity of those pathways is a real challenge for understanding how SOEs participate in cell metabolism and energy production. Recently, the complete sulfite oxidation pathway from *T. thermophilus* has been described [16]; it was shown that the electrons generated during sulfite oxidation are injected into the respiratory chain at the level of cytochromes *c*_552_, via the di-heme cytochrome *c*_550._ Here we have undertaken a detailed investigation of the role played by the novel cytochrome *c*_550_ in linking sulfite oxidation to cell respiration.

Sequence analysis, limited proteolysis and individual expression of the N- and C- terminal regions of the cytochrome confirmed that overall the protein consists of two independent domains, each associated with one *c*-type heme. At physiological pH, the protein is most likely characterized by an asymmetric charge distribution: it has a negatively charged N-terminal domain and a positively charged C-terminal one. The detailed analysis of other di-heme *c*-type cytochromes revealed several features shared by *c*_4_-type cytochromes. Cytochromes *c*_4_ are periplasmic or membrane-bound members of class I cytochromes *c* with a molecular mass of ∼20 kDa found in a variety of bacteria [37], [38]. Analysis of the characterized cytochromes *c*_4_ from *Vibrio cholerae* [39], *Pseudoalteromonas haloplanktis* [40], *Pseudomonas aeruginosa*, *Azotobacter vinelandii* [41], *Acidithiobacillus ferrooxidans* [42] and *Pseudomonas stutzeri* [43], [44], and the high resolution X-ray structures of the last two [45], [46] show that, similarly to *T. thermophilus c*_550_, these di-heme proteins are formed by two domains connected with a flexible, ∼10 aa long linker.

While *T. thermophilus c*_550_ exhibits only ∼15% identity to cytochromes *c*_4_, the latter cytochromes show relatively high sequence similarities not only one each other, but also between the two domains of the same cytochrome (though to a minor extent). This led to the hypothesis that cytochromes *c*_4_ result from duplication of a common ancestral gene [45], [46]. Given the low sequence conservation typically observed among multi-heme cytochromes *c* [47], it would be tempting to postulate close relationships between *T. thermophilus c*_550_ and cytochromes *c*_4_. In the case of cytochrome *c*_550_, however, the fusion of two distantly related cytochromes rather than gene duplication appears to be more likely; the domains indeed exhibit both remarkably different sequences and sizes (with *c*_550_[N] and *c*_550_[C] representing 1/3 and 2/3 of the full-length protein, respectively). It seems very likely that *c*_550_[C] originates from duplication and divergence of cytochrome *c*_552_ [48]. Similarly, fusion of two distantly related cytochromes has been proposed also for the di-heme cytochrome *c* subunit of the flavocytochrome *c* sulfide dehydrogenase from *Chromatium vinosum*. This subunit exhibits ∼15% identity to both cytochromes *c*_4_ [49] and *T. thermophilus* cytochrome *c*_550_, while showing a fold very similar to that of cytochrome *c*_4_ [50].

Despite the low sequence similarity, the dipolar nature of *T. thermophilus* cytochrome *c*_550_ (resulting from an asymmetric charge distribution between the two domains) has been also reported for the cytochromes *c*_4_ isolated from *P. stutzeri* and *A. ferrooxidans* [43], [45], [46]. It has been proposed that the dipolar nature of the cytochrome *c*_4_ from *P. stutzeri* is important for the interaction of the cytochrome with its redox partners. The positively charged C-terminal domain is proposed to interact with the negatively charged pocket of cytochrome *c* oxidase, while negatively charged N-terminal domain with a reductase [45]. However, despite the extensive characterization of *P. stutzeri* cytochrome *c*_4_ (see references in [44]), its specific physiological function remains to be established as yet. Conversely, analysis of the cytochrome *c*_4_ from *A. ferrooxidans* showed the crucial role of a negatively charged residue E121 localized on the overall positively charged C-terminal domain in recognition of its electron donor, rusticyanin, while the Y63 localized in the negatively charged N-terminal domain seems to be responsible for the electron transfer from *c*_4_ to cytochrome *c* oxidase [51].

The results presented here clearly show that the N- (but not the C-) terminal domain of *T. thermophilus* cytochrome *c*_550_ binds to and accepts electrons from SOR_TTHB8_. Based on the 3D model generated for SOR_TTHB8_ and IEX chromatography experiments on *c*_550_[N], recognition between these two proteins most likely involves electrostatic interactions between the positively charged area surrounding the molybdopterin cofactor in SOR_TTHB8_ and the overall negative charge of *c*_550_[N]. Electrostatic interactions have been also proposed to drive the reaction of SorT from *S. meliloti* with its negatively charged natural electron acceptor, cytochrome Smc04048 [20]. The importance of electrostatic interactions between SOR_TTHB8_ and *c*_550_[N] is further supported by the presence of a conserved arginine residue (R50) in the SOR_TTHB8_ in a position that has been identified as crucial for electron transfer in SorA from *S. novella* (R55) [35], [52]. Functionally, therefore, the *c*_550_[N] resembles the heme subunit of a classical Group 1 SOR, and the full-length cytochrome *c*_550_ resembles the cytochrome *c* subunit of the flavocytochrome *c* sulfide dehydrogenase from *Chromatium vinosum*, where the N-terminal part of the di-heme cytochrome *c* tightly interacts with the flavin-containing enzymatically active subunit of the protein.

Our results show that the two domains of cytochrome *c*_550_ can rapidly exchange electrons once the *c*_550_[N] has been reduced by SOR_TTHB8_. Furthermore, both domains are also able to reduce cytochrome *c*_552_, the molecule connecting sulfite oxidation directly to the respiratory chain. The *c*_550_[C] most likely binds *c*_552_ *via* hydrophobic interactions and electron transfer is therefore only modestly affected by the ionic strength. Hydrophobic interactions have also been proposed to be involved in the reaction of cytochrome *c*_4_ from *A. ferrooxidans* [46], [51] with its electron acceptor. Moreover they have also been shown to be particularly important in *T. thermophilus* [30] and in thermophilic organisms in general, as electrostatic interactions are weakened at high temperatures [53].

The finding that both domains of *T. thermophilus c*_550_ can pass electrons to *c*_552_ rules out the possibility that the protein acts as a wire, where one heme serves as the entrance and the other as the exit site for electrons. Our results show that in principle the pathway could be functional with only *c*_550_[N], as this domain is able by itself to shuttle electrons from SOR_TTHB8_ to *c*_552_. The *c*_550_[C] seems, therefore, dispensable for *c*_550_ to fulfill its function. However, the presence of a second heme center in rapid redox equilibrium with both *c*_550_[N] and *c*_552_ enables cytochrome *c*_550_ to assist a two-electron transfer process, similarly to the di-heme subunit of flavocytochrome *c* sulfide dehydrogenase from *C. vinosum* [50]. This confers to cytochrome *c*_550_ the ability to store the two electrons generated during oxidation of a single sulfite molecule and inject them into the terminal oxidases of the respiratory chain *via* cytochrome *c*_552_, thereof ensuring an efficient coupling between sulfite oxidation and the respiration-mediated energy production.

## Acknowledgments

We thank T. Palmer for the *E. coli* TP1000 strain and L. Thöny-Meyer for the plasmid pEC86.
